# Polydatin Inhibits NLRP3 Inflammasome in Dry Eye Disease by Attenuating Oxidative Stress and Inhibiting the NF-κB Pathway

**DOI:** 10.3390/nu11112792

**Published:** 2019-11-15

**Authors:** Bongkyun Park, Kyuhyung Jo, Tae Gu Lee, Soo-Wang Hyun, Jin Sook Kim, Chan-Sik Kim

**Affiliations:** 1 Clinical Medicine Division, Korea Institute of Oriental Medicine, Daejeon 34054, Korea; bkpark@kiom.re.kr (B.P.); berong35@kiom.re.kr (T.G.L.); 2 Herbal Medicine Research Division, Korea Institute of Oriental Medicine, Daejeon 34054, Korea; jopd7414@kiom.re.kr (K.J.); swhyun@kiom.re.kr (S.-W.H.); jskim@kiom.re.kr (J.S.K.); 3 Medicinal Evaluation Team, Gyeonggi Bio-Center, Gyeonggido Business & Science Accelerator (GBSA), Suwon 16229, Gyeonggi-do, Korea; 4 Korean Convergence Medicine, University of Science Technology (UST), Daejeon 34113, Korea

**Keywords:** polydatin, dry eye disease, inflammation, NLRP3 inflammasome, reactive oxygen species, conjunctival cell

## Abstract

Polydatin (also named pieceid, (E)-piceid, (E)-polydatin, trans-polydatin, or 3,5,4’-trihydroxystilbene-3-b-D-glucoside) is a monocrystalline compound isolated from the root and rhizome of *Polygonum cuspidatum* Sieb. et Zucc. (Polygonaceae). A previous study showed that polydatin has antioxidant and anti-inflammatory effects. However, the effect of polydatin in dry eye disease (DED) has not been elucidated. DED rat models were induced by exorbital lacrimal gland-excision. In vivo, the present study showed that the excision of lacrimal glands induced changes such as reduced tear fluid, severe corneal irregularity, damage, tear film break, and goblet cell loss as well as increased inflammation cytokine and NLRP3 expression in conjunctival tissue. However, these changes were restored by polydatin eye dropping. In vitro, polydatin inhibited hyperosmolar stress-induced inflammation through attenuation of the translocation of NF-κB to the nucleus and the mRNA expression of TNF-α, IL-6, IL-1β, and MMP9. In addition, the hyperosmolar stress-induced NLRP3 inflammasome pathway and ROS production were inhibited by polydatin. Our findings provided insight into the effect of polydatin as a candidate reagent for the treatment of DED.

## 1. Introduction

Dry eye disease (DED) is a disorder of the tear glands, ocular surface, and conjunctival tissue. It is caused by increased osmolarity of the tear film and inflammation of corneal and conjunctival tissues [1]. Accordingly, the diagnosis of DED is related to ocular irritation symptoms, such as dryness, soreness, discomfort, and grittiness [2].

The pathogenesis of DED is initiated by aging, hormonal changes, autoimmune disease, and air pollutants. Primarily, dysfunction of the lacrimal gland and Meibomian gland affects goblet cell death in the conjunctiva, resulting in damage to the tear film. The increased osmolarity of the damaged tear film stimulates inflammation responses and induces reactive oxygen species (ROS) production, which triggers apoptosis of corneal and conjunctival epithelial cells [3].

Inflammasomes play a critical role as a regulator of the innate immunity of the host defense against inflammatory diseases [4]. Many studies showed that ROS generation is an essential factor activating NLRP3 (NACHT, LRR, and PYD domain-containing protein 3, nucleotide-binding oligomerization domain-like receptor family, pyrin domain-containing 3) inflammasome [5]. NLRP3, an intracellular danger sensing receptor, is induced by various pathogens and endogenous danger-associated signals [6]. It is mainly expressed in peripheral blood mononuclear cells (PBMCs) and some epithelial cells [7]. NLRP3 inflammasome is also associated with several human autoimmune, inflammatory, and ocular diseases. Moreover, Zheng has reported that ROS generation and inflammasome formation induced dry eye [4]. However, the precise mechanism of the inflammasome in the development of DED is still unclear.

A variety of natural compounds of isolated edible and medicinal plants that report anti-inflammatory effects have been investigated for use as pharmacological candidates. The bioactive constituents of *Polygonum cuspidatum,* such as polydatin, resveratrol, quercetin, and rutin, have various bio-activities that contain antimicrobial, anti-virus, neuroprotective, anti-inflammatory, and cardioprotective effect [3]. Additionally, they have been used in natural cosmetics and medicines and have been showed to have fewer side effects than industrial products. However, there is still no adequate information associated with the health promotion effects of bioactive constituents and the pharmaceutical potential, such as eye health.

**Figure 1:**
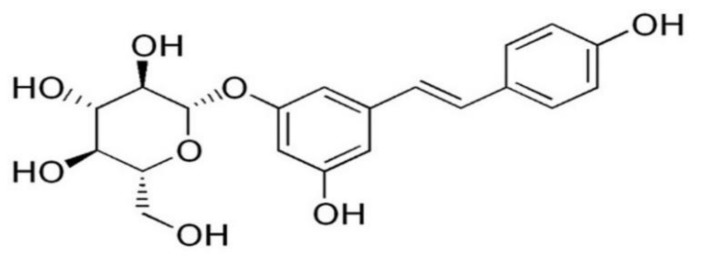
Chemical structure of polydatin.

Polydatin (3,5,4′-trihydroxystilbene-3-β-d-mono-d-glucoside) is a major active component in *Polygonum cuspidatum*. Its chemical structure is shown in Figure 1. In previous studies, polydatin has shown numerous biological and pharmacological effects against human lung cancer, diabetes, shock, ischemia, and respiratory diseases [8,9,10]. Furthermore, polydatin exerts anti-inflammatory, antioxidant, and anti-apoptotic effects against numerous pathogens and pollutants [11]. However, a few previous studies have elucidated the effect of polydatin on DED in vitro and in vivo [12].

In the present study, we examined the effect of polydatin in exorbital lacrimal gland-excised rat models. We mainly focused on the protective effect of polydatin on the ocular surface through inhibition of the NF-κB pathway and NLRP3 inflammasome both in vivo and in vitro.

## 2. Materials and Methods

### 2.1. Reagents

Polydatin was purchased from J&H Chemo Co., Hangzhou, China. RPMI-1640 medium, phosphate-buffered saline (PBS), fetal bovine serum (FBS), and trypsin-EDTA were purchased from Welgene Inc., Korea. Antibodies against NLRP3 (# 13158), phospho-p65 (# 3031), p65 (# 8242), Lamin A (# 4777), α-tubulin (# 2125), COX-2 (# 4842), caspase-1 (# 3866), SOD1 (# 4266), GPx (# 3206), HO-1 (# 43966), and β-actin (# 3700) were purchased from Cell Signaling Technology (Danvers, MA, USA). The ROS inhibitors *N*-acetyl-l-cysteine (NAC) and H_2_DCFDA were purchased from Sigma Chemical Co., St. Louis, MO, USA.

### 2.2. Animals and Treatment

Six-week-old male Wistar rats were purchased from Orient Bio (Seoul, Korea). The DED rat model was established according to previously reported protocols [13]. Rats in the normal control group (NOR) were not subjected to a surgical operation. At three days after surgery, the exorbital lacrimal gland-excised rats were randomly allocated to three groups: (1) DED-induced rats treated with vehicle (DED); (2) DED-induced rats treated with 0.05% polydatin eye drop (polydatin-0.05); (3) DED-induced rats treated with 0.5% polydatin eye drop (polydatin-100); 0.5% polydatin was diluted with 10% polydatin dissolved in 3% Dimethyl sulfoxide (DMSO) by PBS. Polydatin eye drops were administered three times a day. The animal experiments were approved by the Institutional Animal Care and Use Committee (IACUC approval No. 18-028).

### 2.3. Tear Volume Measurement

Tear volume was measured on day 7 after surgical operation. All of the experiment was conducted according to previously known protocols [13]. Phenol red-impregnated cotton threads (Zone Quick, USA) were held with fine forceps and placed in the lateral canthus for 1 min. Tear volume was then measured under a microscope and expressed as the length of the color-changed threads that absorbed the tear fluid.

### 2.4. Analysis of Corneal Irregularity

The corneal irregularity was investigated in rats from each group, as previously described [13]. Briefly, reflected lines of ring-shaped light from the fiber-optic ring illuminator of a stereomicroscope (SZ51; Olympus, Tokyo, Japan) were lighted on the corneal surface of anesthetized rats, and the reflected lines of the light were captured with a DP21 digital camera (Olympus, Tokyo, Japan). Scores of corneal irregularity were graded according to the number of distorted quadrants in the reflected white ring as follows: 0, no distortion; 1, distortion in one quadrant; 2, distortion in two quadrants; 3, distortion in three quadrants; 4, distortion in all four quadrants; 5, severe distortion in which no ring could be recognized.

### 2.5. Ocular Surface Evaluation

All experimental procedures were performed in both eyes of each subject. The data of the eye with the worst damage were collected for analysis. To measure tear breakup time, sodium fluorescein dye was added to the eye, and the tear film was observed under a slit lamp while the rats were prevented from blinking until tiny dry spots developed. Generally, >10 s was thought to be normal [10], 5 to 10 s, marginal, and <5 s, low. To evaluate corneal epithelial defects, the corneas were stained with 3% Lissamine Green (Sigma-Aldrich, St. Louis, MO, USA). The fluorescein score was analyzed as follows: 0, absent; 1, slightly punctate staining in less than 30 spots; 2, punctate staining in more than 30 spots, but without diffusion; 3, severe diffused staining, but without positive plaque; 4, positive fluorescein plaque. Representative images of each scale were provided previously [14].

### 2.6. Histology

To evaluate the density of conjunctival goblet cells, conjunctival sections were stained with periodic acid Schiff (PAS) (Sigma-Aldrich, St. Louis, MO, USA) according to the manufacturer’s instructions. The sections were photographed using a virtual microscope (Olympus, Tokyo, Japan). Goblet cell density in the superior and inferior conjunctiva was measured in three sections of each eye and indicated as the number of goblet cells per 100 μm.

### 2.7. Immunohistochemistry

To investigate the expression levels of NLRP3, immunohistochemical staining was performed according to previously reported protocols [15]. The antibody used in this study was the mouse anti-NLRP3 antibody (Cell Signaling Technology, Danvers, MA, USA). To detect NLRP3, the slides were labeled using a labelled streptavidin biotin (LSAB) kit (DAKO, Santa Clara, CA, USA) and visualized with a DAB substrate kit (DAKO). For morphometric analyses, the immunoreactive intensity per unit area (mm^2^) was measured using the ImageJ software (NIH, Bethesda, MD, USA).

### 2.8. Cell Cultures and Reagents

The human conjunctival cell line HCC was obtained from the American Type Culture Collection (ATCC, Manassas, VA, USA). Cells were cultured according to the manufacturer’s instruction in RPMI medium (Welgene Inc., Korea) supplemented with 100 IU/mL penicillin, 100 mg/mL streptomycin, and 10% heat-inactivated fetal bovine serum (FBS), which was purchased from Thermo Fisher Scienti Waltham, USA, in an incubator at 5% CO_2_ and 37 °C. For sub-culturing, the cells were detached using 0.125% trypsin containing 0.01 M EDTA (Thermo Fisher Scientific, Waltham, USA).

### 2.9. Western Blotting Analysis

HCCs were seeded in 60 mm dishes (5 × 10^5^ cells/mL) and co-treated with polydatin (0.1, 1, or 10 μM) from 1 h to 24 h for each antibody. The cells were washed with PBS and lysed with RIPA lysis buffer (Thermo Fisher Scientific, Waltham, USA). Cell lysate (25 μg) was separated by 8%, 10%, and 12% sodium dodecyl sulfate–polyacrylamide gel electrophoresis (SDS-PAGE) and transferred to polyvinylidene fluoride (PVDF) or nitrocellulose membranes. The membranes were blocked in skim milk dissolved in Tris-HCl-based buffer containing 0.2% Tween 20 for 1 h. The membranes were incubated with the following diluted (1:1000) primary antibodies: COX-2, p65, phopho-p65, Caspase-1, NLRP3, HO-1, SOD-1, GPX, α-tubulin, β-actin, and Lamin A (Cell Signaling Technology, Danvers, MA, USA) in Tris-HCl-based buffer containing 0.2% Tween 20 (pH 7.5). Band intensities were measured using the ImageJ software (NIH, Bethesda, MD, USA).

### 2.10. Nuclear and Cytosol Protein Extraction

Proteins from the nucleus and cytosol were isolated using NE-PER Nuclear and Cytoplasmic Extraction Reagents (Thermo Fisher Scientific, Waltham, MA, USA). Briefly, HCCs were collected with trypsin-ethylenediaminetetraacetic acid (EDTA) and washed twice with PBS. Next, the cells were centrifuged at 15,000 rpm for 10 min, and the supernatants were removed. Ice-cold CER-I and -II solutions (Thermo Fisher Scientific, Waltham, MA, USA) were added per the manufacturer’s instructions to separate cytoplasmic proteins from nuclear-compartment proteins. Western blotting for p65, Lamin A, α-tubulin, and β -actin was conducted.

### 2.11. Quantitative Real-Time PCR

Total RNA was extracted using the single-step guanidinium thiocyanate-phenol-chloroform method. The yield and purity of the RNA were confirmed by measuring the ratio of absorbance at 260 and 280 nm. The isolated RNA (1 mg/mL) was reverse-transcribed using a SuperScript II kit (Bio-Rad, Hercules, CA, USA) for cDNA. The cDNA was subjected to quantitative real-time (qRT)-PCR using specific primers listed in Table 1 by using a thermocycler from Bio-Rad, Hercules, CA, USA.

### 2.12. ROS Production Assay

HCCs (3 × 10^5^ cells/mL) were co-treated with various concentrations of polydatin and hyperosmotic media (528 mOsM) from 30 min to 6 h. The cells were stained for 15 min at 37 °C with 5 μM H_2_-DCFDA (Sigma) on ice in the dark. Each sample was measured for fluorescence intensity using a Cytofluor 2350 (Millipore, USA) and photographed using a fluorescence microscope (Olympus). Changes in the level of intracellular ROS are expressed in comparison with the hyperosmolar stress-induced group.

### 2.13. Statistical Analysis

Representative data from three independent experiments are presented as means ± standard error of the mean (SEM). Comparisons between control and experimental values were analyzed with one-way analysis of variance. Analyses were performed with Prism 7 from GraphPad Software (Graphpad Holdings, LLC, San Diego, CA, USA). Statistical significance was defined as *p* < 0.05.

## 3. Results

### 3.1. Effects of Polydatin on Dry Eye Disease In Vivo

**Figure 2:**
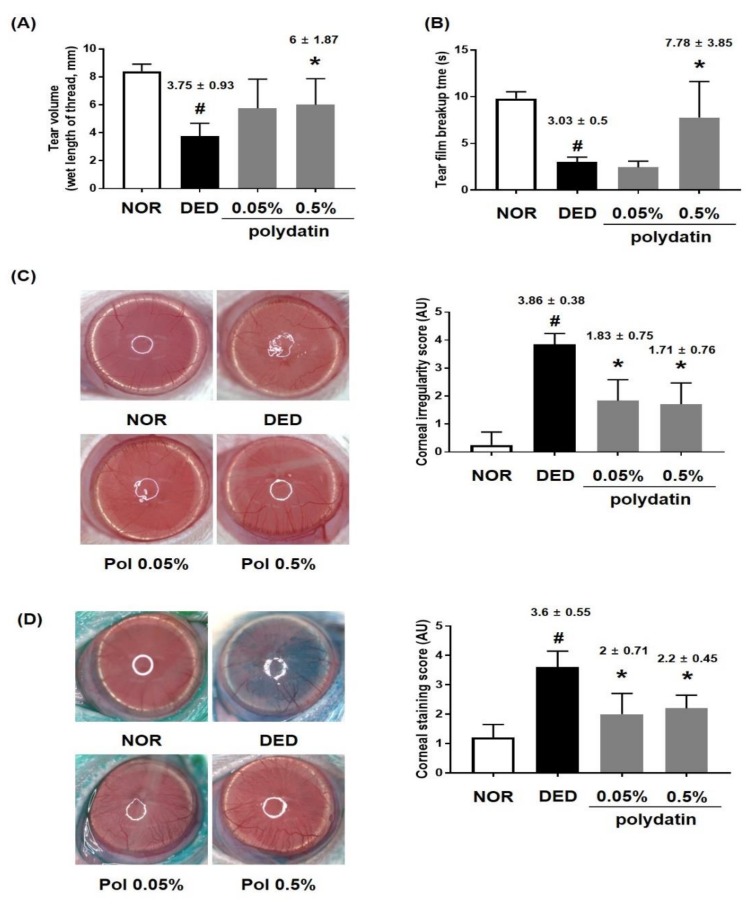
Effects of polydatin in dry eye disease in vivo. (**A**) Tear volume was measured using the phenol red thread tear test. Tear volume was expressed in millimeters of thread that became wet by the tear and turned red. (**B**) Value of tear film breakup time (TBUT) after treatment with polydatin 0.05% and 0.5%. (**C**) Reflected images of a white ring from the fiber-optic ring illuminator of a stereomicroscope. Scale bar is 1 mm; (**D**) Lissamine green staining and its index. The values in the bar graph represent the mean ± standard error (SE), *n* = 7. * *p* < 0.05 vs. normal rats, # *p* < 0.05 vs. vehicle-treated dry-eyed rats.

To investigate the effects of polydatin on DED, we performed in vivo experiments using an exorbital lacrimal gland-excised model. Tear fluid secretion was significantly inhibited through excision of the lacrimal gland (DED), compared to that in the normal group (3.75 ± 0.93 mm, *p* < 0.0001). However, the group treated with 0.5% polydatin showed remarkably restored tear volume, compared to that in the DED group (6 ± 1.87, *p* < 0.01) (Figure 2A). Furthermore, tear film breakup time was considerably short in the DED group (3.03 ± 0.5, *p* < 0.0001) (Figure 2B). The treatment with 0.5% polydatin recovered tear film breakup time in the exorbital lacrimal gland-excised eyes, compared to that in the DED group (7.78 ± 3.84, *p* < 0.005) (Figure 2B). To determine whether polydatin has an alleviating effect on DED-induced corneal tissue damage, the corneal irregularity and staining score were measured. The corneal irregularity was severe in the DED group. However, treatment with polydatin at 0.05% and 0.5% reduced corneal irregularity, significantly decreasing the quantitative score of corneal irregularity (Figure 2C). Corneal staining using Lissamine Green revealed considerable corneal damage in the DED group. In the groups treated with polydatin at 0.05% and 0.5%, the quantitative data were significantly reduced to 2.1 ± 0.71 and 2.2 ± 0.45, respectively, compared to those in the DED group (Figure 2D).

### 3.2. Effect of Polydatin on the Conjunctival Epithelium in Exorbital Lacrimal Gland-Excised Rats

**Figure 3:**
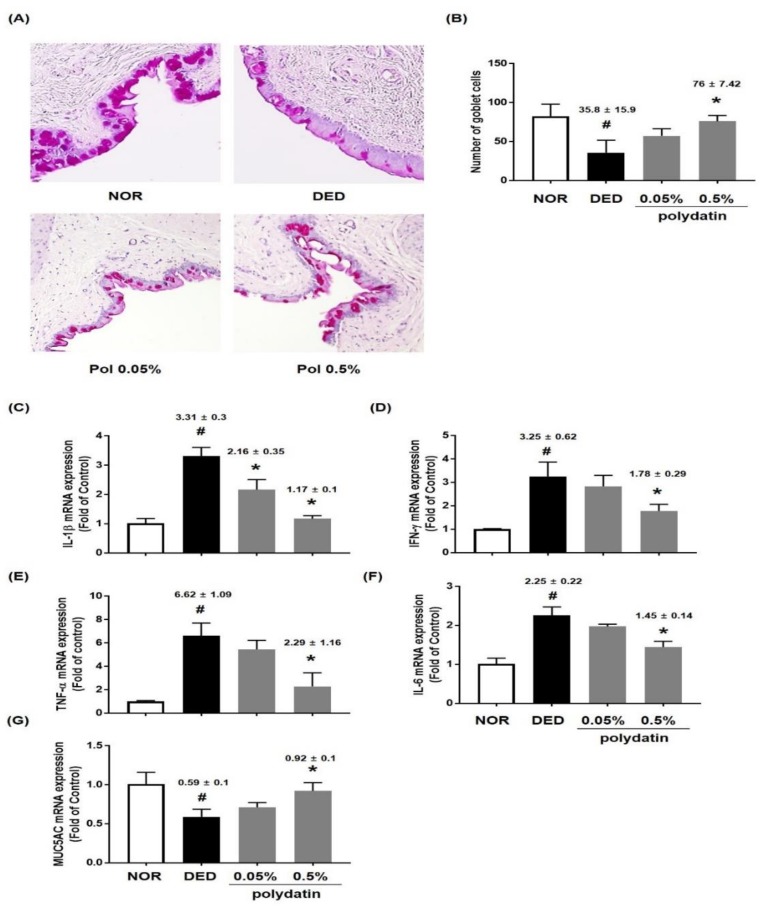
Effects of polydatin on conjunctival goblet cells in exorbital lacrimal gland-excised rats. (**A**,**B**) Histology by Periodic acid-Schiff (PAS) staining of the conjunctival epithelium in exorbital lacrimal gland-excised rats. In the conjunctiva, epithelial cells and subepithelial fibroblasts are seen. PAS positive goblet cells were distributed in the conjunctival epithelium. Bar, 100 µm. mRNA levels of (**C**) IL-1β, (**D**) INF-γ, (**E**) TNF-α, (**F**) IL-6, and (**G**) MUC5AC were assessed by real-time PCR assay. GAPDH was considered an internal control. Data are the mean ± SEM of three independent experiments for all groups. # *p* < 0.05, significantly different from normal mice, * *p* < 0.05, significantly different from vehicle-treated dry eye mice.

The reparative role of polydatin on conjunctival goblet cell loss in the conjunctival tissue of exorbital lacrimal gland-excised rats was examined. We found that treatment with polydatin at 0.5% significantly ameliorated DED-induced conjunctival goblet cell loss (Figure 3A,B). In previous studies, DED has been reported to cause inflammatory reactions in conjunctival tissues [16]. Therefore, qRT-PCR was performed to determine if polydatin has an anti-inflammatory effect on DED-induced inflammation in the conjunctival tissue of exorbital lacrimal gland-excised rats. As shown in Figure 2C,D, the DED group showed a remarkable increase in the mRNA expression of IL-1β, IFN-γ, TNF-α, and IL-6, and decreased mRNA expression of MUC5AC. Treatment with polydatin markedly inhibited the mRNA expression of inflammatory cytokines, but significantly recovered MUC5AC mRNA expression (Figure 3C–G). These data suggested that polydatin might relieve DED by restoring the number of goblet cells through upregulation of MUC5AC mRNA expression and downregulation of the mRNA expression of inflammatory cytokines in conjunctival tissues.

### 3.3. Effect of Polydatin on NLRP3 Inflammasome in Exorbital Lacrimal Gland-Excised Rats

**Figure 4:**
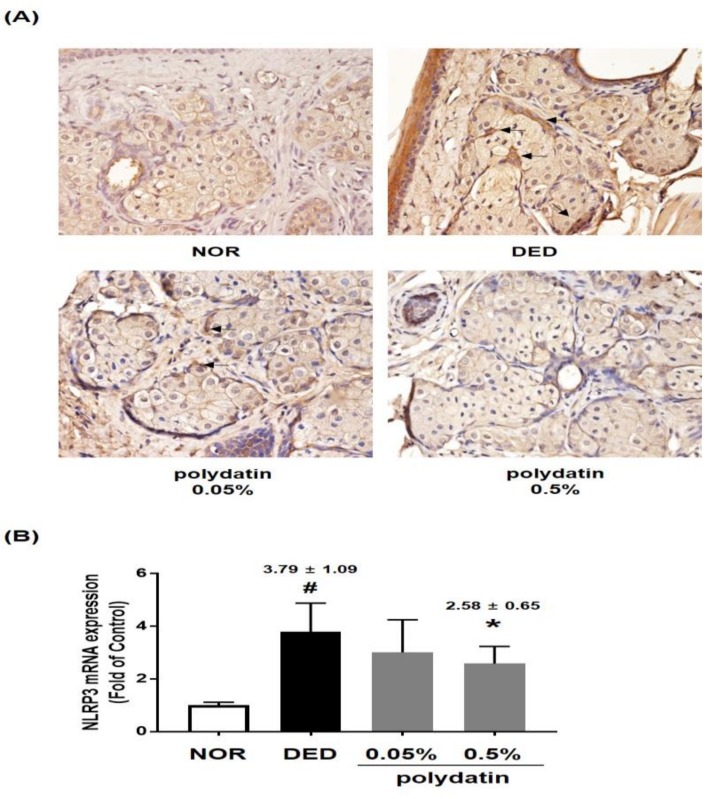
Effect of polydatin on NLRP3 inflammasome in exorbital lacrimal gland-excised rats. (**A**) Immunohistochemical staining for NLRP3. (**B**) mRNA level of NLRP3 was examined by real-time PCR assay. GAPDH was considered an internal control. Data are the mean ± SEM of three independent experiments for all groups. # *p* < 0.05, significantly different from normal mice, * *p* < 0.05, significantly different from vehicle-treated dry eye mice.

It has been demonstrated that NLRP3 inflammasome is increased in human eyes with DED, and its downstream factors, caspase-1, IL-1β, and IL-18, are elevated in patients with DED [17]. Therefore, we examined the effect of polydatin on NLRP3 inflammasome in DED-induced rat conjunctival tissues, and the results are shown in Figure 4. Immunohistological data showed that NLRP3 expression significantly increased in the DED group; however, 0.5% polydatin considerably decreased the mRNA and protein expression of NLRP3 in conjunctival tissues (Figure 4A,B). Taken together, these data suggested that polydatin decreased the expression of inflammatory cytokines by inhibiting the NLRP3 inflammasome pathway in conjunctival tissues.

### 3.4. Effects of Polydatin on Hyperosmolar Stress-Induced Cytotoxicity and Inflammation

**Figure 5:**
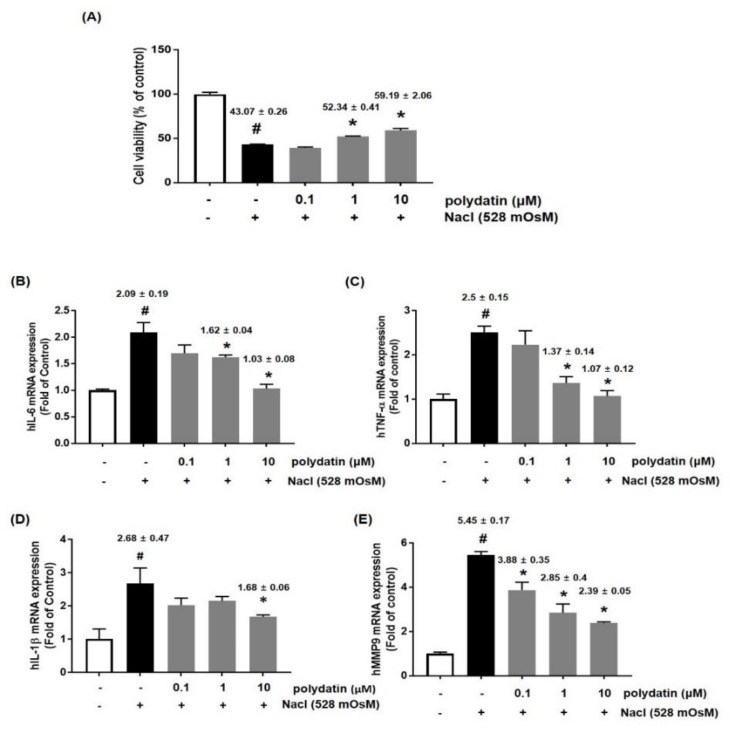
Effects of polydatin on hyperosmolar stress-induced inflammation in human conjunctival cells (HCCs). (**A**) HCCs were co-treated with polydatin (0.1–10 μM) and hyperosmotic media for 24 h. To investigate cell viability, CCK-8 assay was performed. The mRNA levels of IL-6 (**B**), TNF-α (**C**), IL-1β (**D**), and MMP9 (**E**) were assessed by real-time PCR assay. GAPDH was considered an internal control. Data are the mean ± SEM of three independent experiments for all groups. # *p* < 0.05, significantly different from the untreated group, * *p* < 0.05, significantly different from the hyperosmotic-treated group.

**Figure 6:**
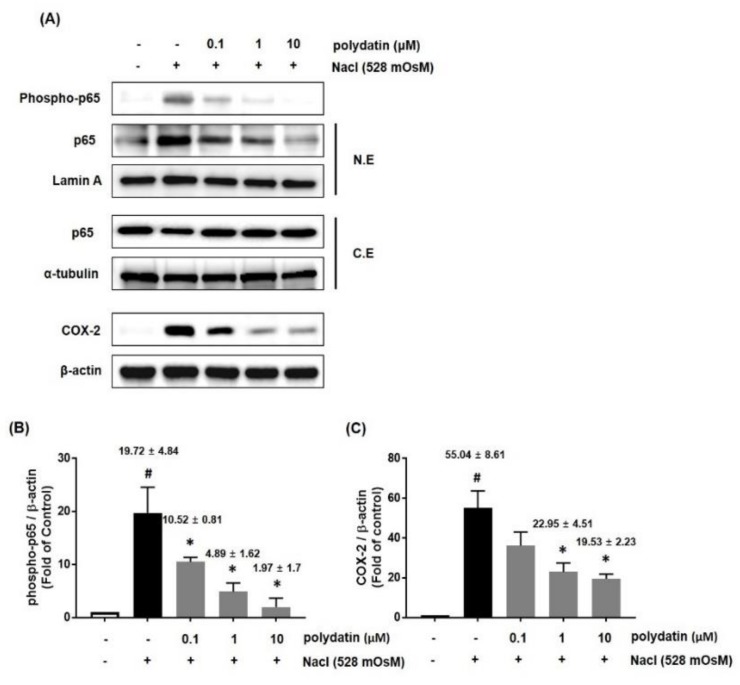
Effects of polydatin on inflammation in hyperosmolar stress-induced HCCs. HCCs cells were co-treated with the indicated concentrations of polydatin and hyperosmotic media (528 mOsM) for 1 h (p65) or 8 h (COX-2). (**A**) The protein expression of phosphor-p65 and COX-2 was analyzed by western blotting analysis. Cytoplasmic and nuclear levels of NF-κB p65 were detected by western blotting to analyze the translocation of NF-κB. α-tubulin and Lamin A were used as loading controls. (**B**,**C**) The relative intensities are expressed as the ratio of phosphor-p65, nuclear p65, and COX-2 to Lamin A or β-actin. Data are the mean ± SEM of three independent experiments for all groups. # *p* < 0.05, significantly different from the untreated group, * *p* < 0.05, significantly different from the hyperosmotic-treated group.

We further confirmed the effect of polydatin on DED in vitro in hyperosmolarity-induced human conjunctival cells (HCCs) by using the CCK-8 assay. The results showed that treatment with NaCl (528 mOsM) for 24 h significantly induced cell cytotoxicity, but polydatin at concentrations of 1 and 10 μM remarkably attenuated the cell cytotoxicity (Figure 5A). To determine whether polydatin regulates the expression of inflammatory cytokines and MMP9, HCCs were treated with the indicated concentration of polydatin in hyperosmotic media for 24 h. qRT-PCR showed that the mRNA expression of IL-6, TNF-α, and IL-1β was significantly increased by the hyperosmotic media, but co-treatment with polydatin markedly attenuated each mRNA expression (Figure 5B–D). In addition, MMP9 mRNA expression was remarkably inhibited by polydatin in a concentration-dependent manner (Figure 5E). We next investigated the effect of polydatin on the inflammatory pathways in hyperosmotic stress-induced HCCs. The protein expression of p-p65 and COX-2 was measured by western blotting. The protein expression of p-p65 and COX-2 was significantly increased by hyperosmotic stress, but treatment with polydatin at the indicated concentration considerably decreased p-p65 and COX-2 expression (Figure 6A,C). Moreover, translocation of p65 to the nucleus was significantly blocked by polydatin in a concentration-dependent manner (Figure 6A,B).

### 3.5. Effect of Polydatin on the Hyperosmolar Stress-Induced Inflammasome Signaling Pathway

**Figure 7:**
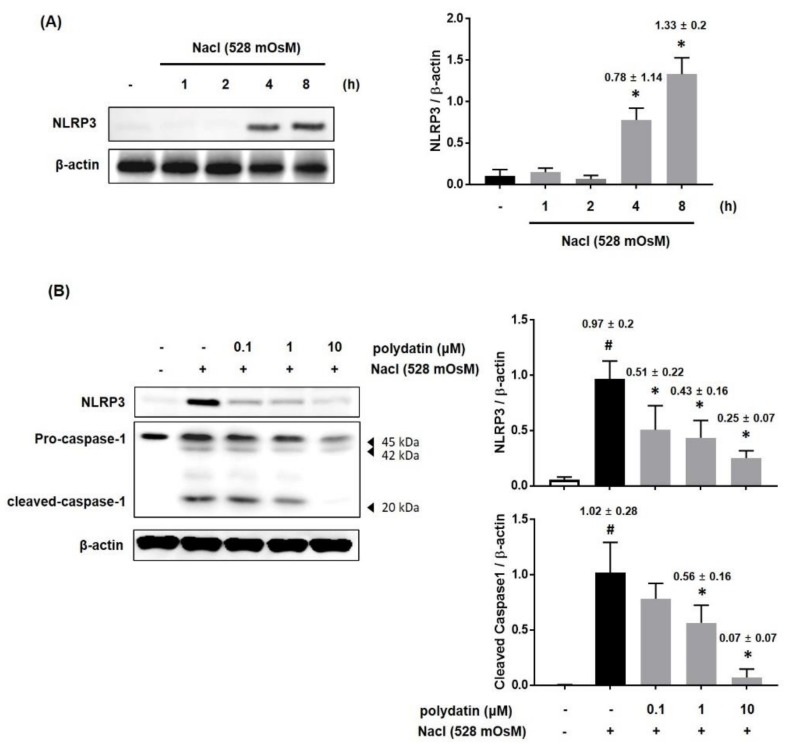
Effects of polydatin on NLRP3 expression in hyperosmolar stress-induced HCCs. (**A**) HCCs were treated to hyperosmotic media (528 mOsM) for each time or 8 h. (**B**) The protein expression of NLRP3 and caspas-1 was analyzed by western blotting analysis. The relative intensities are expressed as the ratio of NLRP3 and cleaved caspase-1 to β-actin. Data are the mean ± SEM of three independent experiments for all groups. # *p* < 0.05, significantly different from untreated group, * *p* < 0.05, significantly different from hyperosmotic-treated group.

It has been demonstrated that NLRP3 inflammasome plays a vital function in the regulation of inflammation and immune response in human DED [17,18]. We next determined whether polydatin modulates the NLRP3 inflammasome pathway in hyperosmolar stress-induced HCCs. We treated HCCs with hyperosmotic cell culture media (528 mOsM) for 8 h. Under hyperosmolar stress, the protein level of NLRP3 markedly increased from 4 h (Figure 7A). Therefore, HCCs were co-treated with polydatin (0.1–10 μM) and hyperosmotic cell culture media (528 mOsM) for 8 h. As shown in Figure 7B, the protein expression of NLRP3 and activation of caspase-1 in HCCs were inhibited by polydatin in a dose-dependent manner, indicating that polydatin regulated the hyperosmolar stress-induced NLRP3 inflammasome pathway (Figure 7B).

### 3.6. Effect of Polydatin on ROS Production in Hyperosmolar Stress-Induced HCCs

**Figure 8:**
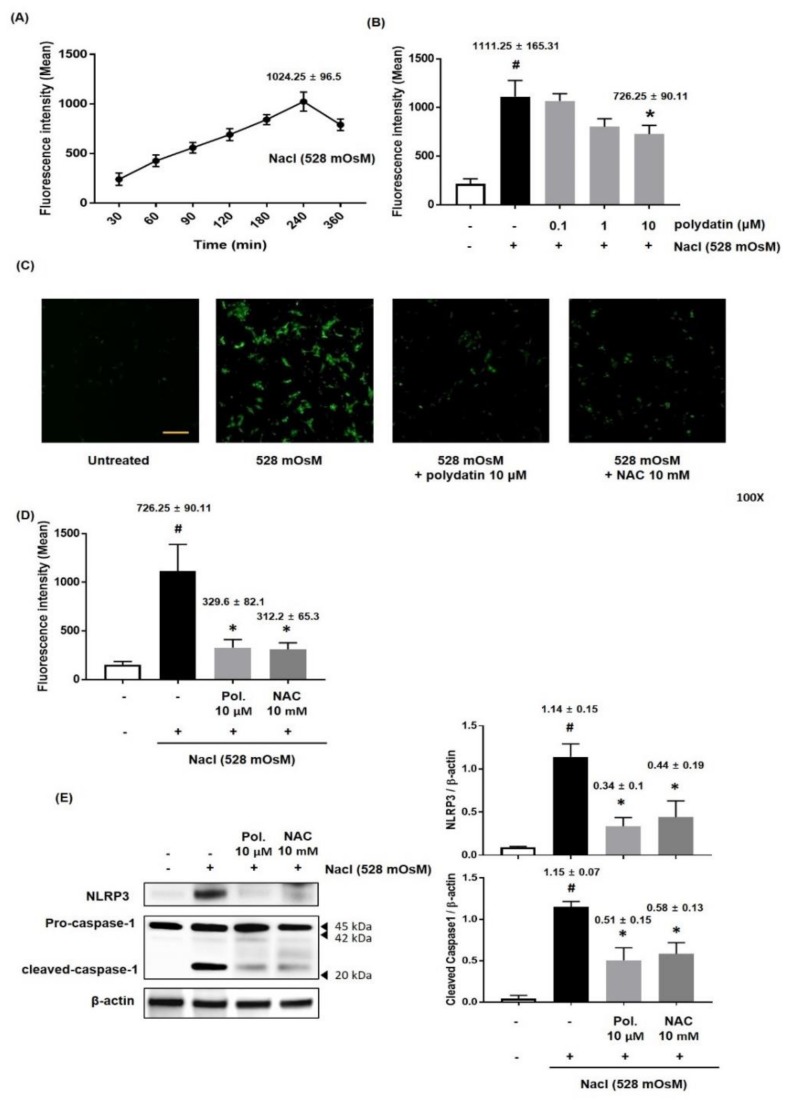

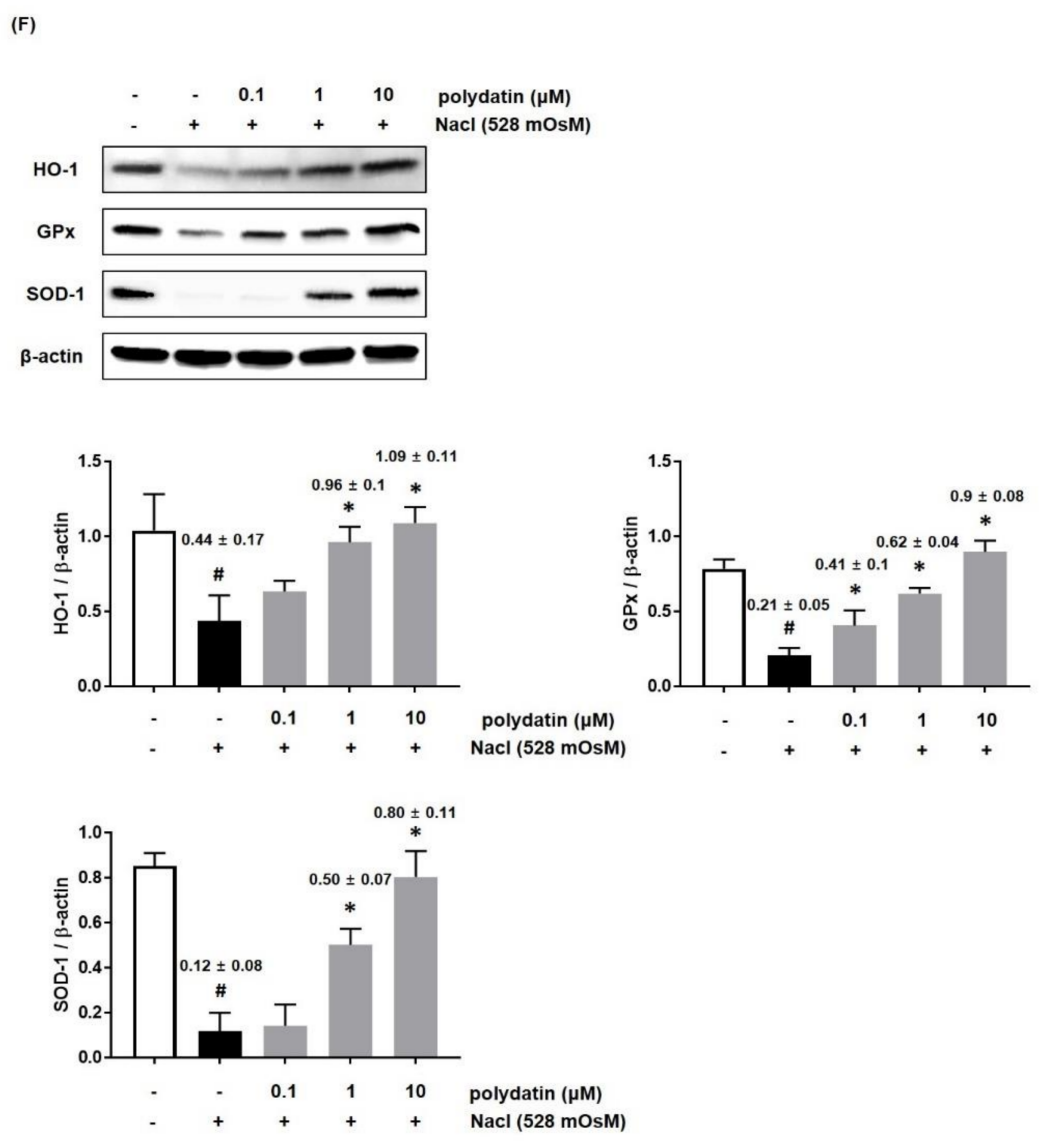
Effects of polydatin on reactive oxygen species (ROS) production in Hyperosmolarity stimulated HCCs. (**A**) Time course graph displayed a time-dependent increase of DCF fluorescence intensity in HCCs exposed to media with 528 mOsM for 30–360 min, respectively. DCF fluorescence intensity was measured by fluorescence microreader. (**B**) The HCCs were co-incubated with different concentrations (0.1–10 μM) of polydatin and Nacl (528 mOsM) to measure DCF fluorescence for 4 h. (**C**) ROS production was observed under a fluorescent microscope at 100× magnification. (**D**) HCCs were co-treated with the indicated concentration of NAC, ROS inhibitor, polydatin, and Nacl (528 mOsM) for 4 h. (**E**) The protein expression of NLRP3 and Caspase-1 using treatment of NAC (10 mM) and polydatin (10 μM) was analyzed by western blotting analysis. The relative intensities are expressed as the ratio of NLRP3, and cleaved caspase 1 to β-actin. (**F**) The HCCs were co-incubated with different concentrations (0.1–10 μM) of polydatin and NaCl (528 mOsM) for 4 h. The protein expression of HO-1, GPx, and SOD-1 was analyzed by western blotting analysis. The relative intensities are expressed as the ratio of HO-1, GPx, and SOD-1 to β-actin. Data are the mean ± SEM of three independent experiments for all groups. # *p* < 0.05, significantly different from the untreated group, * *p* < 0.05, significantly different from the hyperosmotic-treated group.

Numerous previous studies have reported that oxidative stress is implicated in the pathogenesis of DED and that high level of ROS, which is known as a regulator of cell signaling and homeostasis, leads to severe cell damage [19,20,21]. Therefore, we determined the effect of polydatin on hyperosmolar stress-induced ROS production. ROS production continuously increased to 240 min and then slightly decreased to 360 min (Figure 8A). Furthermore, HCCs were co-treated with polydatin (0.1–10 μM) and hyperosmotic cell culture media (528 mOsM) for 4 h. As shown in Figure 8B, hyperosmolar stress-induced ROS production was significantly inhibited by polydatin at the highest concentration tested (Figure 8B). Next, we confirmed the effect of treatment with polydatin and hyperosmotic cell culture media (528 mOsM) for 4 h on the production of ROS compared to that of NAC, a well-known ROS specific inhibitor. Fluorescence assay showed that polydatin remarkably ameliorated ROS production as effectively as the positive control treatment (Figure 8C,D). Moreover, treatment with polydatin or NAC significantly suppressed the expression of NLRP3 and activation of caspase-1 induced by treatment with hyperosmotic cell culture media (528 mOsM) (Figure 8E). We assessed whether polydatin affects the restoration of antioxidant proteins in hyperosmolar stress-induced HCCs. The Western blotting analysis revealed that hyperosmolar stress considerably reduced the expression of HO-1, GPx, and SOD-1 in HCCs. However, the expression of antioxidant proteins was recovered by polydatin in a concentration-dependent manner, suggesting that polydatin exerted an antioxidant capacity to alleviate DED (Figure 8F).

## 4. Discussion

In the present study, we estimate the in vivo and in vitro anti-inflammatory effect of polydatin in exorbital lacrimal gland-excised rats and human conjunctival cell lines. These data demonstrated that attenuation of inflammation by polydatin could be valuable in the treatment of dry eye disease. In vivo, polydatin recovered tear volume and goblet cell density by inhibiting severe corneal irregularity, damage, and tear film break as well as NLRP3 inflammasome. In vitro, polydatin inhibited hyperosmolar stress-induced inflammation through attenuation of the translocation of NF-κB to the nucleus and the mRNA expression of TNF-α, IL-6, IL-1β, and MMP9. Besides, hyperosmolar stress-induced NLRP3 inflammasome pathway and ROS production were inhibited by polydatin.

Polydatin is a stilbenoid glucoside initially obtained from the root and rhizome of *P. cuspidatum,* a folk herbal medicine that has long been used in Asia as a therapeutic agent to treat analgesic, cough, hepatitis, jaundice, amenorrhea, leucorrhoea, arthralgia, hyperlipidemia scalding and bruises, snake bites, and carbuncles [3,22]. In the present study, we showed that treatment with polydatin attenuated DED. In vivo, treatment with polydatin significantly inhibited corneal and conjunctival tissue damage due to exorbital lacrimal gland-excision by restoring tear volume, tear film, and goblet cell density, as well as inhibiting inflammation. In vitro, polydatin treatment suppressed hyperosmolar stress-induced cell cytotoxicity and inflammation via blockage of the NF-κB and NLRP3 inflammasome signaling pathways as well as ROS production.

DED has been shown as a multifactorial illness of the tears and ocular surface characterized by symptoms of dryness and irritation [23]. The ocular surface, which is comprised of the cornea, conjunctiva, lacrimal glands, and connective tissue, can be affected by an imbalance of tear film stability and osmolarity, leading to hyperosmotic damages, such as oxidation, inflammation, and cell apoptosis [24]. Until now, several animal models have been used to investigate DED, but we used lacrimal gland-excised rats to evaluate the effect of polydatin on pathology of DED because lacrimal gland excision allows observation of attenuated tear volume, increased corneal surface irregularity, disrupted corneal epithelial barrier function, reduced conjunctival goblet cell viability, and induced ocular surface inflammation and immunity [25]. The roles of inflammatory cytokines of the ocular surface and conjunctival tissue are also associated with their epithelial dysfunction; IL-1 and IFN-γ expressions lead to squamous metaplasia of ocular-surface epithelial cells, and IFN-γ attenuates goblet cell differentiation [26,27]. In our study, polydatin recovered the tear volume and conjunctival goblet cells by inhibiting the shortening of tear breakup time, corneal surface damage, and mRNA expression of inflammatory cytokines. In addition, it has been shown that induced MMP9 initiates corneal extracellular matrix degradation and epithelial cell loss [28]. Our data showed that hyperosmolar stress induced mRNA expression of MMP9 in HCCs, but treatment with polydatin inhibited MMP9 mRNA expression in a dose-dependent manner.

Nucleotide-binding domain leucine-rich repeats, pyrin domain-containing 3 (NLRP3) is a pattern recognition receptor, and it plays an important role in the signal transduction systems involved in innate immune reactions [29]. Activated NLRP3 by infection or various stress induces apoptosis-associated speck-like protein containing a caspase activation and recruitment domain (CARD) domain (ASC), and then NLRP3 inflammasome is formed in combination with activated caspase-1 precursor. NLRP3 inflammasome induces activation of IL-1β precursor and leads to acute inflammatory responses [30,31]. It has been reported that NLRP3 inflammasome expression and activated IL-1β are increased in DED animal models and that IL-1β concentration is elevated in tears of patients with DED [4]. In the present study, NLRP3 inflammasome was observed in lacrimal gland-excised rats, and NLRP3 expression and caspase-1 activation were significantly increased by hyperosmolar stress. However, treatment with polydatin remarkably inhibited NLRP3 expression in DED-induced conjunctival tissue, caspase-1 activation, IL-1β mRNA expression in hyperosmolar stress-induced HCCs.

It has been known that intracellular ROS plays a crucial role in the regulation of inflammatory responses, and ROS production induced by hyperosmolar stress causes increased activation of the NLRP3 inflammasome, leading to enhanced secretion of the bioactive IL-1β; this indicates that the ROS-NLRP3- IL-1β signaling pathway plays a critical role in environment-induced DED progression. It was also reported that ROS activates various transcription factors, well known as NF-κB, leading to increased NLRP3 protein expression [32]. This suggests that ROS is actively involved in all cellular mechanisms. In our data, hyperosmolar stress induced ROS production after 15 min of treatment, and ROS was highly produced at 240 min of treatment. However, treatment with polydatin significantly attenuated hyperosmolar stress-induced ROS production by recovering the inhibition of antioxidant proteins in HCCs, suggesting that polydatin had pro-antioxidant activity that is as strong as that of NAC, a ROS-specific inhibitor. Based on these findings, the effect of polydatin on NLRP3 expression and NF-κB activation induced by hyperosmolar stress might be attributable to its antioxidant activity and inhibitory effect on ROS production.

## 5. Conclusions

We showed that lacrimal gland excision and hyperosmolar stress led to attenuations of tear volume, shortening of tear film break time, and conjunctival goblet cell damage through corneal and conjunctival damage mediated by enhanced inflammation. However, these changes were restored by polydatin treatment. Additionally, polydatin inhibited hyperosmolar stress-induced NLRP3 inflammasome in HCCs by inhibiting NF-κB signaling pathways and ROS production. Thus, the inhibitory activity of polydatin on NLRP3 inflammasome indicated the possibility of specific pharmacological intervention against inflammatory diseases, including DED. In addition, this study suggests that polydatin is safe to use daily treatment under its doses without any adverse side effects.

## Figures and Tables

**Table 1:** Realtime PCR primer sequences.

Genes	Sequence	
rIL-1β	Sense	5’-CCAGGATGAGGACCCAAGCA-3’
	antisense	5’-TCCCGACCATTGCTGTTTCC-3’
rIL-6	Sense	5’-AGAGACTTCCAGCCAGTTGC-3’
	antisense	5’-AGCCTCCGACTTGTGAAGTG-3’
rTNF-α	Sense	5’-TCGTCTACTCCTCAGAGCCC-3’
	antisense	5’-ACTTCAGCGTCTCGTGTGTT-3’
rMUC5AC	Sense	5’-TCCGGCCTCATCTTCTCC-3’
	antisense	5’-ACTTGGGCACTGGTGCTG-3’
rIFN-γ	Sense	5’-ATCTGGAGGAACTGGCAAAAGGACG-3’
	antisense	5’-CCTTAGGCTAGATTCTGGTGACAGC-3’
rNLRP3	Sense	5’-ATCTGGAGGAACTGGCAAAAGGACG-3’
	antisense	5’-CCTTAGGCTAGATTCTGGTGACAGC-3’
rGAPDH	Sense	5’-GGGACTCAAGCTCCTCTGTG-3’
	antisense	5’-GAGGCTCTGGTTATGGGTCA-3’
hIL-1β	Sense	5’-AATCTGTACCTGTCCTGCGTGTT-3’
	antisense	5’-TGGGTAATTTTTGGGATCTACACTCT-3’
hIL-6	Sense	5’-AAATTCGGTACATCCTCGAC-3’
	antisense	5’-CAGGAACTGGATCAGGACTT-3’
hTNF-α	Sense	5’-TTCTCCTTCCTGCTTGTG-3’
	antisense	5’-CTGAGTGTGAGTGTCTGG-3’
hMMP9	Sense	5’-GGGACGCAGACATCGTCATC-3’
	antisense	5’-TCGTCATCGTCGAAATGGGC-3’
hGAPDH	Sense	5’-CCAGCCGAGCCACATCGCTC-3’
	antisense	5’-ATGAGCCCCAGCCTTCTCCAT-3’
